# Co-producing a ‘creative toolkit’ to support the mental health and wellbeing of palliative care professionals: a community case study

**DOI:** 10.3389/fsoc.2025.1488840

**Published:** 2025-02-14

**Authors:** Marie A. Clancy, Caitlin R. Kight, Jessica Stein, Naome Glanville, Anthony C. Wilson, Richard G. Kyle

**Affiliations:** ^1^Academy of Nursing, Department of Health and Care Professions, University of Exeter, Exeter, United Kingdom; ^2^School of Education, University of Exeter, Exeter, United Kingdom; ^3^Hospiscare, Exeter, United Kingdom; ^4^Arts & Culture, University of Exeter, Exeter, United Kingdom

**Keywords:** creativity, art, palliative care, wellbeing, mental health, nursing, COVID-19

## Abstract

Alterations to the clinical, social, and economic landscape have made palliative care an increasingly challenging sector in which to work; COVID-19 introduced further changes that pushed palliative care professionals to the breaking point. Their struggles at work are exacerbated by the fact that specialists in this field tend to ignore their own needs, instead centring and prioritising those of their patients—a situation that is not tenable. Within this community case study we describe how our team, comprising clinical and university staff, sought to address this by co-creating a suite of resources to support the physical, psychological, social, and spiritual health of palliative care workers. The result was the Creative Toolkit©, which is both an overall approach and a suite of materials that uses creative, arts-based intervention to facilitate reflection, relaxation, and rejuvenation—and to ensure that clinical staff feel connected and valued. Although participants sometimes initially hesitate due to their limited prior exposure to art-based methods, feedback on our sessions has been unanimously positive, revealing the value of creative activities for, among other things, aiding in processing difficult feelings and creating community amongst staff. While initial results are promising, we acknowledge the need for an expanded evidence base to encourage more widespread uptake of our method.

## Introduction

Worldwide, palliative care places a notable emotional burden on professional caregivers and, as a result, requires innovative methods of support to allow practitioners to practice safely and effectively. Palliative care has been understood in many different ways, making it difficult to compare, discuss, or agree on good practice. To address this, the Lancet Commission on Global Access to Palliative Care and Pain Relief (Knaul et al., 2018)—an international team affiliated with the World Health Organisation (WHO)—sought to determine a definition that might be applied internationally, to facilitate the implementation of the recommendations from the Commission. The result was an understanding of palliative care as ‘the active holistic care of individuals with serious health-related suffering and especially of those near the end of life’. It was also recognised that palliative care interventions aim ‘to [relieve] suffering and [improve] quality of life for patients and families dealing with any type of life-threatening illness (WHO, 2022). Palliative care teams are recognised as comprising ‘doctors, nurses, support workers, pharmacists, social workers, physiotherapists and volunteers all working together with the patient and their family’ (WHO, 2022). Although the commission also asserts a need to consider the quality of life of people providing palliative care (Knaul et al., 2018; Radbruch et al., 2020a), neither this concept nor its implementation are fully addressed.

There is a wealth of evidence that providing palliative support to patients can negatively impact on the physical, psychological, social, and spiritual health of carers (Cross, 2019). A further challenge is the lack of differentiation between informal caregivers/carers and health care professionals, both of whom may not adequately care for themselves when immersed in the suffering of others. Moreover, as well as the blurring of roles, palliative care interventions are often non-active and therefore the ‘work’ of palliative care professionals is not adequately recognised or valued. This means that the expectations of the support palliative care professionals can provide, and how this should be achieved, can be unclear (Radbruch et al., 2020a).

A review of key models of palliative care practice (Dobrina et al., 2014) found that, among other things, professionals are asked to be:

- ‘present’—not just physically located in a room, but also mentally and emotionally tuned in to the patient such that they can anticipate needs and respond meaningfully and authentically (Reed, 2010)
- non-judgmental (Desbiens et al., 2012)
- reflective and aware of how their own beliefs and actions impact on others (Reed, 2010; Desbiens et al., 2012)
- capable of providing therapy and hope to others while considering their own ongoing growth and transformation (Hutchings, 2002; Murray, 2007; Reed, 2010; Desbiens et al., 2012).

This requires a commitment to sharing oneself openly in interactions where pain, suffering, and, ultimately, loss are present (Dobrina et al., 2014). Caring relationships where there is close proximity to distress and confrontation with mortality may create and compound distress among palliative care professionals. Palliative care nurses, in particular, report high levels of compassion fatigue, death anxiety, moral distress, and burnout (Batson, 2017; Cross, 2019; Hussain, 2021).

In the United Kingdom, as globally, palliative care professionals tend to ignore their own needs to centre and prioritise those of their patients (Watson et al., 2019), and often use unhelpful coping mechanisms in response to the stresses of the job (Whitebird et al., 2013; Parola et al., 2017). Although there have been reports and initiatives that specifically highlight the need for nurses to balance their own needs against those of their patients (Marie Curie, 2017; Macmillan Cancer Support, 2017; NHS England, 2019)—i.e., recognising that one ‘cannot pour from an empty cup’—it is all too common for guidance about the future of palliative care to focus primarily on the patient and the process, without considering the staff who will bring those goals to life (e.g., Schroeder and Lorenz, 2018). This is not to say that palliative care is an inherently negative specialism; many nurses report that their jobs are fulfilling and rewarding (Wallerstedt and Andershed, 2007). However, it can be incredibly challenging for palliative care professionals to maintain good mental health and wellbeing. Poor physical and mental health is often associated with a rise in errors, sickness absence, and premature workforce exit—all of which threaten the long-term viability of palliative care services (Health Education England, 2019).

### Palliative care professionals in the post-pandemic era

These challenges have become particularly noticeable since the COVID-19 pandemic, which significantly impacted the provision of palliative care (Radbruch et al., 2020b; Knights et al., 2024; Bailey et al., 2023). In addition to huge increases in the number of patients requiring support, palliative care nurses also noted that patients were younger, often referred in poorer health, and further along the dying process. Overwhelmingly, the focus shifted to immediate physical care and away from the other three dimensions of palliative support (psychological, social, and spiritual)—with negative consequences not only for those receiving care, but also those providing it (Bailey et al., 2023).

Put plainly, the pandemic has had an indelible effect on palliative care professionals who were already at breaking point (Hussain, 2021). It is now vital to reflect on what initiatives and interventions might allow us to address both the negative impact of the pandemic and the pre-existing issues that it exacerbated. Our goal should not be simply to roll back the clock, but to create a fresh vision of how palliative care professionals could provide quality patient care while also protecting their own wellbeing. However, this is not a straightforward task, nor one that can be achieved through a single initiative focussed on individuals alone. Rather, it requires systemic and structural change, likely underpinned by new policy, practice, and research. A focus on innovative and creative experimentation with small-scale solutions could present a path towards this ambitious goal. If found to be effective these could be shared, scaled, and sustained to facilitate a grassroots, community-led shift in palliative care practice.

### A community case study approach

This paper utilises a community case study approach to document our local experience of co-developing a resource to meet the identified needs of the palliative care community. Specifically, the Creative Toolkit© aims to support the physical, psychological, social, and spiritual wellbeing of palliative care professionals through creative interventions. The Creative Toolkit© is a suite of activities and approaches (described further below) that use art to facilitate reflection, communication, connection, and catharsis. It is an evolving and carefully curated collection of activities that can be used by individuals or groups, offering suggestions for bite-size and/or asynchronous delivery as well as longer and more communal sessions. The Toolkit comprises both face-to-face sessions delivered by members of the project team, as well as a website^1^ that allows users to access and download materials independently.

Among other things, the Creative Toolkit© has been used to support clinical supervision of palliative care staff, structure away days and retreats for palliative care teams, facilitate individual reflections by palliative care providers, and provide guided activities undertaken by nurses and death doulas with their patients.

Although Creative Toolkit© activities continue to be delivered primarily by the original project team, who work to tailor interventions for specific groups, train-the-trainer approaches are being developed to help local leads feel confident in introducing these methods to support their teams. We are also embedding Creative Toolkit© methods into nurse education at the University of Exeter^2^ to grow a new generation of palliative care staff who are equipped to protect their own wellbeing, and that of their colleagues, from their first day on the job.

## Context

The Creative Toolkit(C) Project was conceived, developed, delivered, and evaluated jointly by team members from Hospiscare and the University of Exeter.

### Hospiscare

Hospiscare is the local palliative care charity for Exeter, Mid, and East Devon, a region in the United Kingdom (UK) covering an area of over 1,000 square miles. Every year, Hospiscare delivers support to around 2,500 people living with terminal illness, as well as supporting National Health Service (NHS) colleagues. Patients are cared for in their own homes as well as in a dedicated inpatient unit in Exeter. This is achieved by more than 300 Hospiscare staff and 400 volunteers.

Hospiscare receives 80% of its funding from the local community. The charity has a mission ‘to provide compassionate, expert, end-of life care before, during and after death’, underpinned by the values of ‘Compassion, Respect, Professionalism, and Inclusivity’.^3^ These ambitions are set against the changing landscape of palliative care, demographic challenges of an aging population, increasingly complex care needs, and reduced NHS support services. This has led to increased demands on the charity in recent years, while funding and expert staff have become harder to access, recruit, and retain.

The organisation was represented in the project team by Jessica Stein, nurse educator.

### University of Exeter

The University of Exeter is a Russell Group (research-intensive) university with approximately 30,000 students and 7,000 staff. It comprises three Faculties, two of which are represented in The Creative Toolkit© project team: the Faculty of Health and Life Sciences, which is home to the Academy of Nursing, and the Faculty of Humanities and Social Sciences, which is home to the School of Education.

This organisation was represented in the project team by four academic staff (Marie Clancy, Caitlin Kight, Richard Kyle, Anthony Wilson), one professional services staff (Naome Glanville), and three student interns. Several members of the project team (MC, CK, AW, NG)) contribute to nursing education through the ‘Art and History of Nursing’ module (see below), on which they lead (MC) and contribute specialist knowledge (CK, AW, NG) on health and arts-based learning.

## Methodological detail

### Development stage one: inspiration

The Creative Toolkit© initiative was inspired by teaching techniques used by one of the project leads in her module ‘The Art and History of Nursing’ (Clancy et al., in preparation). Briefly, the module aims to facilitate a holistic approach to nursing by supporting students to develop skills such as observation, empathy, and communication with diverse audiences through arts-based learning. Student evaluation revealed additional benefits of the approach, including positive effects on their own mental health and wellbeing when examining and creating artistic pieces: *“Allows me to think freely, to deeply reflect about certain situations, develop coping mechanisms to certain situations that are suited to me, my needs, hobbies and things that give me joy”*.

Similar benefits were reported by attendees at the two end-of-module exhibitions (Arts & Culture, 2025) at which students had an opportunity to share their work with the public. While this outcome was unforeseen, it was also not surprising. Several studies link creative practice such as visual, musical and theatrical arts with positive mental health and wellbeing (e.g., Forgeard and Eichner, 2014; Wright and Pascoe, 2015; Acar et al., 2020; Tan et al., 2021; Scott, 2021). This is also consistent with the experiences of several members of the project team, who have personally benefitted from engaging with creativity to support wellbeing (e.g., Wilson, 2012; Clancy, 2017; Kyle et al., 2023; Kight and Lemon, 2024; Clancy et al., in preparation).

### Development stage two: inception

Initially, the University-based members of the project team reached out to Hospiscare with the goal of building on the success of the module by helping student nurses use creative techniques to reduce stress during their placements—a ‘real world’ application of the skills developed in the classroom. However, the initial conversation with Hospiscare revealed a need to think more ambitiously. Hospiscare staff shared their organisational experiences of the problematic working conditions in contemporary palliative care and the associated increased strain on clinical staff working to provide support. Given their high workloads, the staff rarely had time to take breaks or decompress—including in the context of clinical supervisions, which had essentially been discontinued despite their known benefits (Bégat and Severinsson, 2006; Ali, 2017, cited in Hussain, 2021).

### Development stage three: iterative interventions and findings

Collectively, we thus developed a new vision for our project—one that prioritised the lived experience of palliative care providers and used co-creation techniques to support these professionals and develop resources for use by them and their colleagues. A small-scale pilot co-led by nursing students for community palliative care nurses suggested our idea was promising. After less than an hour of experimenting with found poetry and collage, all participants agreed that it had been cathartic and rejuvenating to use art as a means of processing the various challenges of the day.

Based on this success, we scaled up our intervention, targeting all staff at Hospiscare. We circulated a short survey (accompanied by a brief video of the pilot event: https://youtu.be/BY0bL5ofzkk) to gather data on:

1. whether and to what extent staff were open to learning and employing creative techniques in professional contexts (quantitative data)
2. what sorts of techniques were of interest (qualitative descriptive data)
3. what sorts of delivery formats would be accessible / preferred (qualitative descriptive data)
4. emotional and mental responses to the workshop (qualitative descriptive data)

Almost unanimously, quantitative data revealed participants felt more comfortable with creative techniques and the possibility of using them in a work environment in the future (post-workshop). However, the qualitative descriptive data gathered, when thematically analysed using Braun and Clarke’s (2022) approach, revealed some scepticism about the methods we were suggesting—a mixture of concerns about the time commitment required, the (perceived) requirement for pre-existing artistic skills, and uncertainty about the ultimate value of the exercises. However, staff were also cautiously curious, and had no trouble volunteering a number of potential creative activities that they would be interested in exploring.

Drawing on recommendations made in the surveys, we subsequently organised two catered half-day retreats that were open to all Hospiscare staff and volunteers—one in the morning and one in the afternoon, to accommodate different working patterns. To further accessibility and pilot different approaches, retreats were held on each of the two Exeter-based university campuses (subsequent retreats have been held elsewhere to facilitate nature-based activities). Each retreat featured three different creative interventions (collage, poetry-writing, and clay work), followed by a group reflective discussion (on each activity, specifically, and on the event as a whole).

Members of The Creative Toolkit© project team collected field notes throughout each event, capturing the actions and attitudes of participants. We also used pre- and post-session questionnaires to gather more quantitative data on the effect of the retreats on participants’ attitudes towards creative approaches. Responses were unanimously positive: Participants shared how the retreats introduced techniques that could be used for their own wellbeing but also that of their patients. Moreover, being given the time and space to engage with creative approaches led to feelings of value and worth among Hospiscare participants which were counter to their initial depleted morale and motivation before the project commenced. Participants also appreciated how the events offered an opportunity to socialise and reconnect with colleagues.

Participants valued their new-found awareness of the range of diverse creative approaches available to them. They wanted further, more frequent opportunities to explore the benefits of these activities in more depth. However, whilst attendees generally agreed that half- or whole-day workshops appeared to be an ideal format, they acknowledged that it might be difficult, given resource constraints, for the organisation to run these sessions as frequently as they might like.

Consequently, to explore alternative options for delivery—including different locations, durations, topics, and formats of the Creative Toolkit© sessions—we ran a variety of additional workshops for a range of audiences. Although participants were from different professions, they were united in being frontline workers in high-pressure environments, responsible for the pastoral care of others. Examples of these additional events included:

- A relatively unstructured one-hour team bonding session at an away day for University of Exeter Wellbeing staff; participants could choose to engage with one of several activities (e.g., rock painting, decorating mugs, drawing mandalas) for which supplies were laid out in stations around the room;
- Lunchtime professional development presentations (both in-person and online, of varying length) for university educators looking to learn specific creative techniques (e.g., sketchnoting, collage) that could be used in tutoring, teaching, or personal self-care;
- One-hour online breakfast discussions for clinical care staff generally interested in learning about, and connecting with colleagues who use, creative techniques in healthcare contexts;
- A structured two-hour development workshop (incorporating techniques associated with naming emotions, expressing gratitude, and using metaphors to describe work challenges) held on-site for a nursing team at a busy metropolitan hospital.

As at the original retreats with Hospiscare staff, participants were invited to contribute to the ongoing evolution of the project by sharing reflections on their experiences and suggesting modifications. Overall responses were similar to the earlier events. For example, participants noted how the activities: facilitated ‘positive thinking in a relaxed way’; provided ‘distraction when stressed or overwhelmed’; helped ‘you think clearly’.

Attendees were interested in how we might make the activities more easily accessible and widely available. Suggestions for additional resources and means of facilitation included: posters (e.g., to hang in restrooms and public spaces in healthcare facilities), public exhibitions, contributions to staff newsletters (e.g., short videos or infographics), training for clinical staff in leadership positions (e.g., internal trainers, clinical supervisors), and a website.

## Discussion

Since the inception of the Creative Toolkit© project, we estimate that we have reached an audience of at least 2,500 people between June 2023 and July 2024:

- 500 participants attending some two dozen workshops—with attendance ranging from five to 30 people and an average of 12 people at each event;
- 800 attendees across physical and virtual exhibitions;
- 500 views of our web activity (two blogs hosted on University of Exeter pages; social media activity of session facilitators and participants; The Creative Toolkit© website);
- >700 delegates at conferences (e.g., European Association for Palliative Care; EduExe) where we have contributed posters and workshops.

Participants sometimes requested minor adjustments to our approach—suggesting, for example, that we incorporate different methods; run events at different times; or change the layout of the room. However, feedback about the Creative Toolkit© initiative *on the whole*—i.e., about the use of creative activities to support reflection, rest, and rejuvenation—was unanimously positive, with participants saying that it was: ‘really valuable to get into the headspace of non-judgemental creativity’; ‘…a “grounding” and “restorative” experience’; ‘like [putting on] an oxygen mask before the plane goes down’.

We received similarly upbeat responses regardless of whether we were delivering online or in person, to palliative care nurses, other clinical staff, educators, or general public audiences. While it is true that sessions were always modified slightly to suit the audience and context, the input of Hospiscare staff was always at the core of every session. This clearly evidences the power and flexibility of a co-design approach (Slattery et al., 2020). It also highlights the incredible wealth of knowledge held by our palliative care participants—who are clearly not just experts in pain and death, as per the common public perception (Patel and Lyons, 2020), but also in care, compassion, and healing.

### Impact of the Creative Toolkit© initiative on Hospiscare

For Hospiscare, the Creative Toolkit© project has been a lynchpin in rejuvenating staff support initiatives designed to protect and prioritise wellbeing in the face of increasing workplace pressures. Having seen the positive effects of the project, Hospiscare are moving towards the idea of being a ‘creatively fluent’ organisation which is well versed in diverse methods of reflecting on palliative care work and rising to its challenges. Working with death and dying has distinctly creative and counter-cultural elements, which means that Hospiscare staff are well placed to optimise innovative, artistic methods.

Participants in the initial Creative Toolkit© activities reported three main benefits: improvement to their own personal wellbeing, an ability to gain perspective and reflect on their work, and learning techniques that could be used in the context of patient care. The Hospiscare Learning and Development Team has initially focused on providing offerings most directly associated with the first two of these, but have often found that interventions are helpful across all three domains.

**Figure 1:**
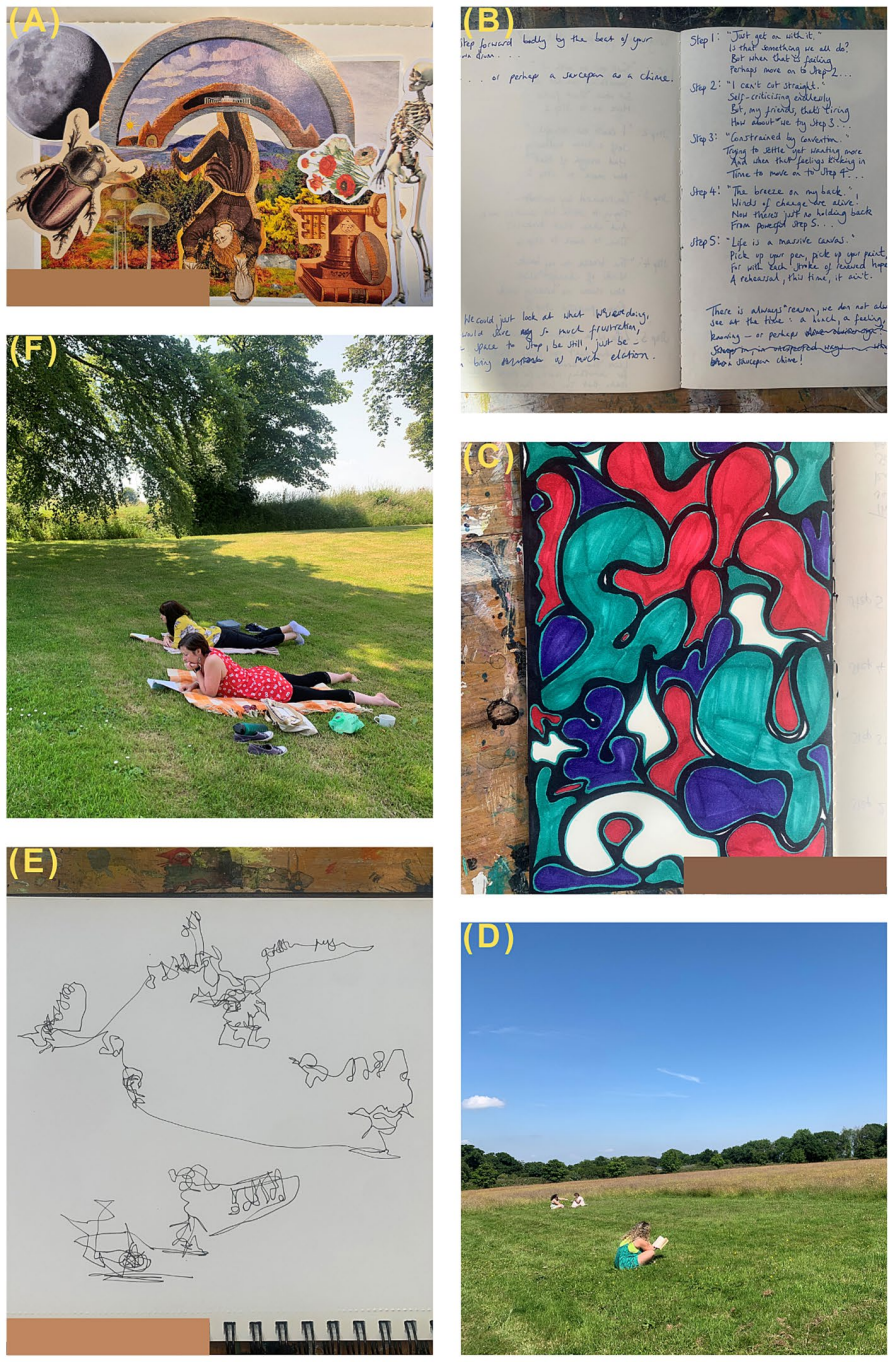
Photographs taken during the inaugural Hospiscare ‘Rest and Relaxation’ day: **(A)** collage made by one of the facilitators; **(B)** reflective journalling produced by a participant; **(C)** drawing produced by a participant during a facilitated creative session; **(D)** clinical staff birdwatching, socialising, and reading during a free period; **(E)** mindful nature sketch made during a facilitated creative session; **(F)** participants reading after the picnic lunch.

There are three specific initiatives that Hospiscare has put in place as a direct result of engaging with the Creative Toolkit© project:

1. At their Annual Updates event, attended by all Nursing and Allied Health professional staff (n > 100 people to date), we introduced a creative reflection exercise—an example of which can be found here: https://youtu.be/G71dQyOQLCA. Feedback was overwhelmingly positive, with staff often identifying it as the most useful part of the day. In line with our experience in the co-led pilot student nurse sessions, participants who initially expressed resistance to the idea of creativity later indicated how effective and enjoyable they found the exercise. One senior member of staff shared that she was pleasantly surprised who at how efficiently the technique enabled staff to get to the heart of how they were feeling.
2. Hospiscare built on this success with a series of more in-depth sessions. At the time of writing, they are mid-way through a series of 6-monthly ‘Creative Clinical Supervision Sessions’ at which a variety of creative methods are used to facilitate discussion and learning on clinical situations. The aim of these events is to create, evaluate, and eventually disseminate a new model of creative supervision that will facilitate a ‘creative fluency’ in supporting the workforce. This directly responds to the need to reinstate good clinical supervision, which staff have missed in the post-COVID era (for example, one person said: ‘I think it is a shame that we have not been able to meet so often, I have missed it! At the time we are most busy, is when we need it most’). This is also in keeping with published recommendations to provide flexible supervision that can support reflection and, via reflection, resilience (Bégat and Severinsson, 2006; Ali, 2017; Hussain, 2021).
3. Hospiscare have also created a series of ‘Rest and Reflection Days’, which focus directly on the wellbeing benefits of creativity. These are designed to allow staff from across the organisation to attend a day-long retreat with a range of optional creative activities (e.g., collage, mindful drawing, painting) as well as opportunities to connect with one another (Figures 1A–F). The first day, which was run in Summer 2024, was universally appreciated by attendees, who welcomed the ‘freedom of the day’, ‘having the choice to join in or do your own thing’, and the fact that it was ‘not too structured’ but still ‘encouraged creativity’. In providing staff with options and the opportunity to express agency in choosing their own activities for the day, we provided a flexibility increasingly rare in the palliative care sector; because of changes in the clinical, social, and economic landscape, these same staff have been experiencing increasing limitations and restrictions during their normal working hours, so one benefit of the Rest and Reflection Day was a chance to regain some autonomy. Participants also highlighted how much they appreciated the ‘wonderful investment and gesture that management appreciate what we do’. This underlines how effective wellbeing offers such as this are in the support and retention of staff—an area that is an ongoing challenge across the clinical landscape (NHS England, 2023). The initial session was at 100% capacity and staff are already eagerly anticipating future sessions. In addition to expressing appetite for further unstructured creative opportunities within the organisation, staff have also showed initiative by, e.g., establishing a monthly craft group after work. We hope the Creative Toolkit© will continue to be a catalyst and encouragement for similar formal and informal activities.

Collectively, the three initiatives sparked by the Creative Toolkit© project have been well received by participants who ‘welcome [the sessions] as a way of making space in their busy days, a time that they can escape from the challenges of work for a few moments.’ Creativity has been found to be particularly useful in this sensitive area of work because it allows difficult subjects to be approached in subtle and indirect ways. For example, one of the most common objections to any form of reflection is that it will ‘open up a can of worms’ (McTaggart et al., 2014, p. 60). Often, reflection sessions take place within the working day and amongst colleagues, so participants can be wary that if they share too much, there will be a negative consequence for them and others (e.g., patients and colleagues). However, creative methods of reflection offer a form of expression which appears to protect participants’ boundaries and confidentiality.

Nevertheless, there is ongoing work required to shift the narrative that creativity can be a waste of time that ‘takes us away from clinical work.’ However, as we continue to offer these initiatives, the benefits are being recognised by the wider team and there is increasing engagement. For instance, one particularly fruitful avenue for future exploration and innovation is the potential to incorporate these methods to enhance patient care. Perhaps unsurprisingly, given the patient-centred nature of the role, staff immediately recognised the potential application of The Creative Toolkit© techniques in clinical interactions. In fact, practitioners seemed to value the Toolkit workshops even more when they could see how the methods could be used in their work: Nurses discussed how creative approaches offered ‘amazing skills to use with patients, particularly those [experiencing] psychological stress’. They also acknowledged that creative approaches were ‘a good way to have a conversation with someone without the pressure’ that comes with direct eye contact.

## Acknowledgment of constraints and limitations

Despite the promising early outcomes from the Creative Toolkit© project, there are four main constraints to be addressed through future experimentation and evaluation. First, it is difficult to quantify the impact of our interventions, both in terms of gauging the effect on attitudes, morale, and wellbeing and in terms of assessing the long-term effect. Second, given current demands on healthcare resources in the UK context, including both staff time and finances, demonstrating the scalability of this intervention is vital. Third, given current reliance on relatively small-scale funding source sustainability of the project needs to be established. Fourth, a key priority is ensuring that resources and events sharing the Creative Toolkit© approach are accessible, inclusive, and culturally sensitive. These are all areas that we are currently exploring through ongoing co-creation with our ever-expanding network of collaborators and participants; this includes international collaborations with colleagues from three additional continents. This cross-cultural co-development will also allow us to better understand replicability and what conditions may be necessary to adopt and adapt our approach in different contexts.

## Data Availability

The raw data supporting the conclusions of this article will be made available by the authors, without undue reservation.

## Footnotes

^1^ https://www.creativetoolkit.online/

^2^ https://www.exeter.ac.uk/study/undergraduate/courses/nursing/

^3^ https://www.hospiscare.co.uk/
